# Surface Modulation of Graphene Oxide for Amidase Immobilization with High Loadings for Efficient Biocatalysis

**DOI:** 10.3390/biom11101399

**Published:** 2021-09-23

**Authors:** Kongliang Xu, Bin Wang, Chenlu Si, Chaoping Lin, Renchao Zheng, Yuguo Zheng

**Affiliations:** 1 Key Laboratory of Bioorganic Synthesis of Zhejiang Province, College of Biotechnology and Bioengineering, Zhejiang University of Technology, Hangzhou 310014, China; kongliangxu@zjut.edu.cn (K.X.); 2111905083@zjut.edu.cn (B.W.); 201706021620@zjut.edu.cn (C.S.); linchaoping@zjut.edu.cn (C.L.); zhengyg@zjut.edu.cn (Y.Z.); 2 Engineering Research Center of Bioconversion and Biopurification of Ministry of Education, Zhejiang University of Technology, Hangzhou 310014, China

**Keywords:** amidase, enzyme immobilization, graphene oxide, high loading, biocatalysis, surface modulation

## Abstract

As a type of important and versatile biocatalyst, amidase immobilization on solid materials has received broad attention with its relatively easy procedure and available reusability. However, current porous supports have suffered from limited loadings, and it is highly desired to develop a new type of material with abundant space so as to ensure a high loading of amidase. Here, graphene oxide was adopted as the support for amidase immobilization, which showed the highest loading capacity for amidase (~3000 mg/g) to date. To the best of our knowledge, it is the first case of amidase immobilized on graphene oxide. Through surface modulation via reducing the contents of oxygen-containing functional groups, activity recovery of immobilized amidase increased from 67.8% to 85.3%. Moreover, surface-modulated graphene oxide can efficiently uptake amidase under a wide range of pH, and the maximum loading can reach ~3500 mg/g. The resultant biocomposites exhibit efficient biocatalytic performance for asymmetric synthesis of a chiral amino acid (i.e., L-4-fluorophenylglycine, an intermediate of aprepitant).

## 1. Introduction

Amidases or amide hydrolases (EC 3.5.1.X) are a type of important versatile biocatalyst, which have been employed in the production of various chiral amino acids, carboxylic acids and amides via the cleavage of C-N bonds [1]. With the increasing demand of green chemistry, amidases have received broad attention in pharmaceutical and chemical industries for the production of D- or L-amino acids and β-lactam antibiotics due to their high activity and specificity under mild conditions [1,2]. However, the intrinsically fragile nature of enzymes under incompatible conditions (e.g., high temperature, unfavorable pH and organic solvents) greatly limits the industrialization of amidases [3]. To improve their stability and recyclability, various methods have been developed, such as enzyme immobilization, protein engineering and so on [4]. In the past decades, immobilization of amidases on solid materials has received broad attention with the relatively easy procedure and available reusability [2], in which the selection of solid support is critically important as the materials have strong influence on the resultant properties. In search of an ideal support for amidase immobilization, various materials with different compositions, structures and morphologies have been explored [5], including inorganic carries (e.g., mesoporous silica) [6], organic carries (e.g., chitosan and nanocrystalline cellulose) [7,8], composite carriers (e.g., silica nanoflower and metal–organic framework hybrids) [9], etc.

Among several features for an ideal host matrix, high loading capacity is one of the important indexes used to evaluate immobilization efficiency [10]. A support with higher loading capacity means less usage of supports and is favorable to obtain a high apparent activity, which will reduce additional costs of the carriers and increase reaction rate [2,11]. In this content, a series of porous materials with high specific surface area and porosity have been investigated for amidase immobilization, such as mesoporous silica [6], polymers [12], etc. In order to further improve the loading capacity, macropores or mesopores were introduced into the porous supports to provide more spaces or sites for amidase accommodation [5]. For example, macropores were introduced into the mesoporous silica spheres, affording a loading capacity as high as 895 mg/g for amidase immobilization [11]. Our group recently prepared hierarchically porous MOFs, which significantly enhanced the loading capacity of amidase on MOFs [13]. Recently, the EziG platform based on porous silica beads seems to be a universal platform for enzyme immobilization, which offered some outstanding performance results when applied in various areas [14,15]. However, current porous supports still suffered from limited loadings of amidase (<1000 mg/g), as enzyme molecules cannot easily penetrate into the internal pores. Therefore, it is highly desired to develop a new kind of materials with abundant space so as to ensure a high loading of amidase.

Recently, graphene oxide (GO), a kind of single-layer two-dimensional (2D) nanomaterials, has received considerable attention due to their large specific areas and abundant functional groups [16]. It is envisioned that graphene oxide can serve as an ideal support for amidase immobilization, as the flat surface can provide abundant spaces or sites and the functional groups on the surface could easily be modulated to regulate the enzyme activity and stability [17,18,19]. Although there are some cases of other enzymes (such as horseradish peroxidase, glucose oxidase, lipase, etc.) immobilized on GO [20,21,22,23,24,25], immobilization of amidases on GO has not been reported yet and their catalytic performance needs to be investigated [5,19].

In this contribution, graphene oxide was prepared and used as the support for amidase immobilization for the first time, which showed the highest loading capacity for amidase to date. To improve the enzymatic activity recovery of immobilized amidase, GOs with different surface properties have been achieved via reducing the contents of oxygen-containing functional groups, leading to an increased activity recovery of 85.3%. Through surface modulation, GOs can efficiently take up amidase under a wide range of pH and the maximum loading can reach ~3500 mg/g. The resultant biocomposites exhibit efficient biocatalytic performance for asymmetric synthesis of a chiral amino acid (i.e., L-4-fluorophenylglycine).

## 2. Materials and Methods

### 2.1. Materials

Graphite flakes (A.R., 100 mesh, Shanghai Aladdin Biochemical Technology Co., Ltd., Shanghai, China), KMnO_4_ (A.R., Beijing Chemical Factory, Beijing, China), NaNO_3_ (A.R., Beijing Chemical Factory), concentrated sulfuric acid (H_2_SO_4_, A.R., Beijing Chemical Factory) and alcohol (CH_3_CH_2_OH, A.R., Beijing Chemical Factory) were used as received. Ultrapure water (resistance > 18 MΩ cm^−1^) was purified by a Sartorius AG arium system.

### 2.2. Instruments and Characterization

Transmission electron microscopy (TEM) images were obtained with a JEM-1010 transmission electron microscope (Hitachi, Tokyo, Japan) operating at 200 kV. Scanning electron microscopy (SEM) images were collected on a JSM-7610F field emission scanning electron microscope (Hitachi, Japan). UV-Vis absorption spectra were taken in a quartz cell with light path of 1 cm on a SHIMADZU UV-2500 spectrophotometer (200–800 nm). Powder X-ray diffraction (XRD) was performed on an X’TRA diffractometer (ARL, Lausanne, Switzerland) and the data were collected from 5° to 80° (D/max rA, using Cu Kα radiation at a wavelength of 1.542 Å). Fourier transform infrared (FT-IR) spectra were collected on a Thermo Nicolet 380 spectrometer (Waltham, WI, USA) using KBr pellets (32 scans), and the spectra were recorded at a resolution of 4 cm^−1^. The circular dichroism (CD) spectra of free amidase and immobilized amidase were acquired by CD spectroscopy (Chirascan Applied Photophysics, Leatherhead, UK) at 25 °C, in which the samples were scanned from 190 to 260 nm. For details, the enzyme samples were diluted into 0.2 mg/mL in phosphate buffer (pH = 7.0), and 200 μL of each diluted solution was transferred to a 0.5 mm path length quartz cuvette, and placed in the sample compartment of CD spectroscopy for the measurements. Zeta potentials of amidase (0.5 mg/mL) as a function of pH were obtained on a Malvern Zetasizer HS-3000 instrument (USA) at 25 °C.

### 2.3. Amidase Expression and Purification

Recombinant amidase was expressed in *Bacillus subtilis* WB800, in which the synthesized gene (from *Bacillus megaterium* CA4098, AF161313.1) were cloned into plasmid pPZW103 for the sequent transformation. *B. subtilis* WB800/pPZW103-PGA was cultivated on LB agar medium containing 50 μg/mL kanamycin. The colony was picked to be inoculated in the medium and cultivated for 24 h. The overexpressed recombinant amidase was isolated by centrifugation and purified through cell-free extraction using ammonium sulfate at 80% saturation, DEAE-IEX chromatography and butyl HIC chromatography. The protein concentration was measured by a bicinchoninic acid (BCA) assay kit with bovine serum albumin as a standard.

### 2.4. Preparation and Surface Modulation of GO

GO was prepared using natural graphite flakes by a modified Hummers’ method according to the previous literatures [26,27,28]. In a typical procedure, 0.75 g NaNO_3_ was added to 34 mL concentrated H_2_SO_4_, followed by the addition of 1 g graphite at 0 °C. Then, the mixture was oxidized by 5 g KMnO_4_ with vigorous mixing, in which KMnO_4_ should be slowly added. The solution was stirred for 2 h after increasing the temperature to 35 °C and then diluted by 50 mL ultrapure water at 0 °C. After being stirred for another 2 h, 4 mL H_2_O_2_ was slowly added and stirred vigorously until no gas was generated. The mixture was then centrifuged and washed with 1:10 aq. HCl (500 mL) and ultrapure water (500 mL). To completely wash the graphite oxide flakes, the resultant graphite oxide was dissolved in water again and centrifuged several times to reach pH 7. The graphite oxide flakes were sonicated in water (70 W) for 5 h, and finally centrifuged to collect a GO solution from a supernatant.

Surface modulation of GO was achieved by the reduction in GO sheets with L-ascorbic acid at room temperature and the degree of reduction was controlled by the reaction time. Briefly, the reduction was performed by adding l-AA (200 mg) to an aqueous dispersion of the GO (2 mg mL^−1^, 50 mL). With the prolonging of the reaction time, GO with less oxygen-containing groups could be obtained, in which GO-3, GO-6 and GO-12 represent GO treated with L-ascorbic acid for 3 h, 6 h and 12 h, respectively. After the reduction was complete, the solid was isolated by filtration and washed with ultrapure water until the pH of the solution was 7.0.

### 2.5. Immobilization of Amidase on GO

Enzyme immobilization was carried out by adding the desired amount of GO to a 0.1 M phosphate buffer (pH = 7.0) of amidase (0.34 mg) at 4 °C, affording the biocomposite denoted as amidase@GO. The mixture was incubated for 1 h with shaking and then centrifuged. The supernatant was used to determine the enzyme loading. The immobilized enzymes were washed three times with the phosphate buffer to remove unadsorbed amidase. The loading capacity of GO for amidase was determined by detecting the concentration of amidase in the supernatant solutions before and after immobilization, in which the unadsorbed amidase in the buffer after being washed three times was taken into account when calculating the total unimmobilized enzyme on GO.

### 2.6. Enzymatic Activity Assay

Kinetic resolution of racemic N-phenylacetyl-4-fluorophenylglycine to produce L-4-fluorophenylglycine (an intermediate of aprepitant) was chosen as a representative reaction to demonstrate the catalytic activity of amidase. The activity of the immobilized/free amidases was measured at 40 °C in 100 mM glycine-sodium hydroxide buffer (pH = 9.0) containing 20 mM substrate (i.e., N-phenylacetyl-4-fluorophenylglycine) and determined by calculating the rate of the L-4-fluorophenylglycine production released from the initial substrate. The reaction was stopped by NaOH and neutralized by HCl. The concentration of L-4-fluorophenylglycine was measured by high-performance liquid chromatography (HPLC) (Shimadzu Co., Kyoto, Japan) with a C18 column (5 μm × 250 mm × 4.6 mm, Welch Materials, Inc., Shanghai, China) using acetonitrile—0.1% perchloric acid (50:50, *v/v*) as the mobile phase at a flow rate of 1.0 mL min^−1^. One unit (U) of enzyme activity was defined as the amount of enzyme that synthesizes 1 μmol of L-4-fluorophenylglycine per minute under the above activity assay conditions.

## 3. Results and Discussion

**Figure 1:**
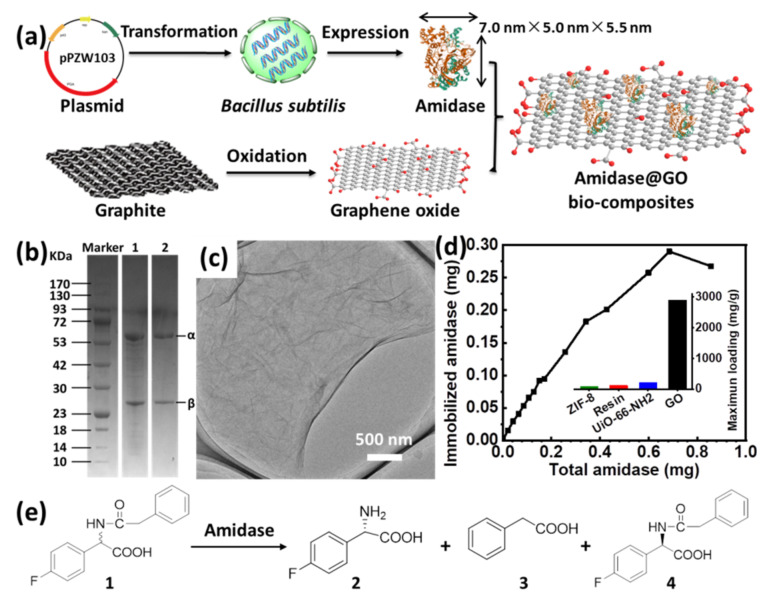
(**a**) Schematic illustration for expression of recombinant amidase, preparation of GO and immobilization of amidase on GO; (**b**) SDS−PAGE of semi-purified (lane 1) and final purified (lane 2) amidase; (**c**) TEM image of GO; (**d**) immobilized amidase on GO as a function of the total amount of amidase and the comparison of maximum loading capacity of amidase on various supports (inset); (**e**) schematic illustration for kinetic resolution of racemic N-phenylacetyl-4-fluorophenylglycine (1) to produce L-4-fluorophenylglycine (2) by amidase.

Schematic illustrations for expression of recombinant amidase, preparation of GO and immobilization of amidase on GO are shown in Figure 1a. Recombinant amidase (7.0 nm × 5.0 nm × 5.5 nm) was expressed in *Bacillus subtilis* WB800 according to our previous reports [29], in which the synthesized gene (from *Bacillus megaterium* CA4098) was cloned into plasmid pPZW103 for transformation. The overexpressed recombinant amidase was isolated and purified, which gave two bands (approximately 26 and 61 kDa) on sodium dodecyl sulfate-polyacrylamide gel electrophoresis (SDS−PAGE) (Figure 1b and Figure S1). Graphene oxide was prepared by a modified Hummers’ method using natural graphite flakes as the precursors (see supporting information for details) [26,27], which was evidenced with the increased interlayer spacing by X-ray diffraction (XRD) spectra (Figure S2). Transmission electron microscopy (TEM) and scanning electron microscopy (SEM) images revealed that as-prepared GO displayed typical 2D nanosheet morphology with ultrathin thickness (Figure 1c and Figure S3).

Amidase immobilization was carried out by adding the desired amount of GO to a 0.1 M phosphate buffer of amidase (pH = 7.0) at 4 °C, affording the biocomposite denoted as amidase@GO. The resultant biocomposite was then washed by phosphate buffer to remove loosely adsorbed amidase. The amidase loading capacity of GO was obtained via a bicinchoninic acid (BCA) assay using UV−vis spectroscopy to detect protein concentration in the supernatant solutions before and after immobilization. To evaluate the maximum amidase loading capacity of GO, immobilized amidase on a certain amount of GO (0.1 mg) as a function of the total amount of amidase was measured (Figure 1d), in which the amidase immobilized gradually increased with the increase in total added amidase. From the above experiment, the maximum amidase loading capacity of GO can reach as high as 2902 mg g^−1^, which is much higher than the materials we have reported before (inset of Figure 1d), such as ZIF-8 (90.5 mg/g) [13], resin (120 mg/g) [30], UiO-66-NH_2_ (211.2 mg/g) [13]. Moreover, to the best of our knowledge, it is the highest loading capacity for amidase immobilization compared with the most efficient supports reported by other groups (Table S1) [7,8,9,11,12,31,32]. The high loading capacity of amidase on GO can be ascribed to the unique structural properties of 2D materials, in which flat surface with abundant functional groups can provide enough spaces or sites for amidase accommodation [16,20].

**Figure 2:**
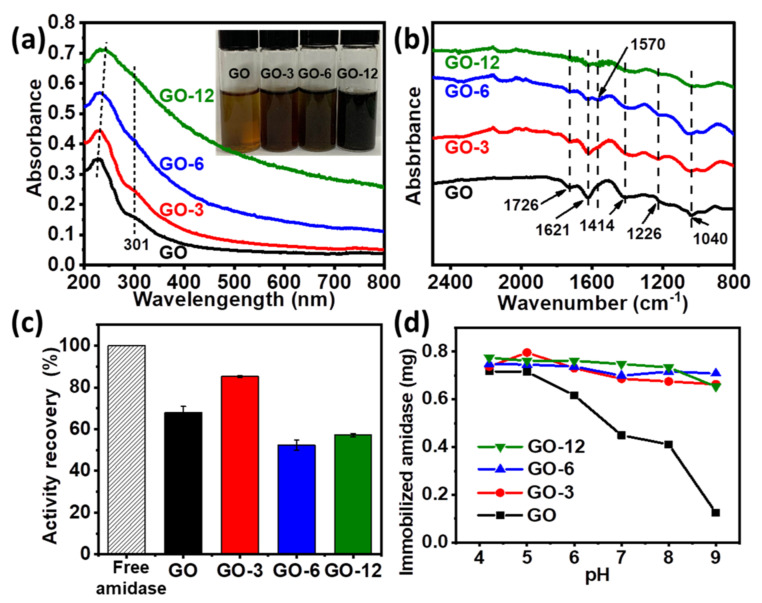
(**a**,**b**) UV-vis spectra (**a**), photograph (inset in (**a**)) and FT-IR spectrums (**b**) of GO, GO-3, GO-6 and GO-12; (**c**) relative activity of free and immobilized amidase; (**d**) effect of pH on the amidase loadings on GO, GO-3, GO-6 and GO-12.

Considering the importance of homochiral amino acids, kinetic resolution of racemic N-phenylacetyl-4-fluorophenylglycine to produce L-4-fluorophenylglycine (an intermediate of aprepitant, Figure S4) was chosen as a representative reaction to demonstrate the catalytic activity of amidase (Figure 1e), in which aprepitant is a medication used to prevent chemotherapy-induced nausea and vomiting [33]. The activity of amidase was evaluated in glycine-sodium hydroxide buffer (pH = 9.0) at 40 °C. However, only 67.8% activity recovery of amidase@ GO was observed after immobilization (Figure S5 and Figure 2c), which might be ascribed to negative effects of cationic residues on GO surface [17,20]. Thus, it is necessary to modulate the surface of GO to achieve a more suitable immobilization environment for amidase accommodation.

Surface modulation of GO was achieved by controlling the contents of oxygen-containing groups (e.g., hydroxyl, carboxyl, carbonyl and epoxy groups) as previously described, which could be gradually reduced by L-ascorbic acid (see supporting information for more details) [20]. With the prolonging of reaction time, GO with less oxygen-containing groups could be obtained, in which GO-3, GO-6 and GO-12 represent GO treated with L-ascorbic acid for 3 h, 6 h and 12 h, respectively. Modulation process was monitored by UV-vis and Fourier transform infrared (FT-IR) spectroscopy. As shown in Figure 2a, the peak of GO at 227 nm (π-π* transition of aromatic C=C) gradually shifted to 230 nm, 234 nm and 243 nm for GO-3, GO-6 and GO-12 and the shoulder peak at around 301 nm (n-π* transition of C=O) gradually disappeared [20,24]. Meanwhile, the color of GO changed from yellowish brown to black (GO-12) as reaction proceeded (inset of Figure 2a). FT-IR spectrums in Figure 2b show that GO has several strong characteristic peaks at 1726 cm^−1^ (C=O stretching vibration in carboxyl or carbonyl groups), 1414 cm^−1^ (O–H deformations in the C–OH groups), 1226 cm^−1^ (C–OH stretching vibration), 1040 cm^−1^ (C–O stretching vibrations in C–O–C of epoxide) and 1621 cm^−1^ (absorbed H_2_O molecules) [24,34]. The peak intensity of oxygen functional groups decreased with the modulating time, and a skeletal vibration absorption peak of C=C at about 1570 cm^−1^ was observed, indicating that partial aromatic structure might be restored when oxygen-containing groups were removed from the GO surface. The above observations, together with XRD and energy dispersive spectrometer (EDS) analysis results (Figure S6 and Table S2), all proved the successful modulation of GO.

Through surface modulation, activity recovery of immobilized amidase on GO-3 increased from 67.8% to 85.3% (Figure 2c), which can be attributed to less oxygen-containing groups on the surface, bringing less negative effects to the enzyme [17]. The relative high activity recovery of amidase on GO can be attributed to less effects to the enzyme activity sites by physical adsorption compared with that immobilized via covalent interactions [13,35,36]. However, further reducing of oxygen-containing groups resulted in a decreased activity (Figure 2c and Figure S7), suggesting that a structural conformation change of amidase may occur on GO-6 and GO-12 and secondary structure of immobilized amidase is partially lost. It was observed that loadings on GO dramatically dropped (Figure 2d) when pH was above the pI value (pI of amidase is determined to be ~4.3, Figure S8). However, enzyme loadings on GO-3, GO-6 and GO-12 were barely affected by pH, suggesting that enzyme loading on modulated GO is insensitive to pH and GO can efficiently uptake amidase under a wide range of pH through surface modulation, and the maximum loading capacity of GO-12 can reach ~3500 mg/g (Figure S9). A high loading amount of amidase on the GOs is favorable to obtain a high apparent activity of the immobilized enzyme. On the other hand, a too high enzyme loading amount is unfavorable for obtaining a high specific activity due to the enzyme overloading, as revealed by our experimental observations (Table S3). Thus, a proper loading amount of amidase (170 mg/g) was used for further investigation.

**Figure 3:**
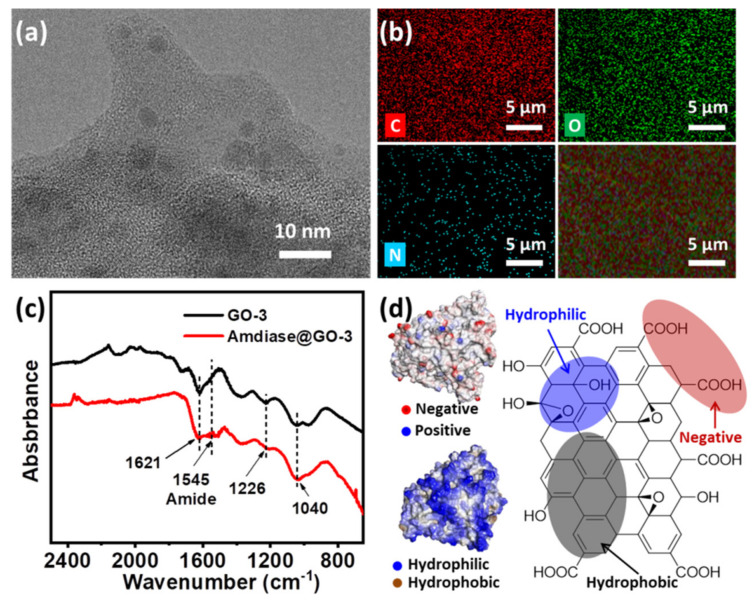
Characterization of amidase@GO-3 biocomposite. (**a**) TEM image; (**b**) elemental mapping images; (**c**) FT-IR spectrums; (**d**) simulated surface property of amidase (left) and structure of graphene oxide (right).

From the above results, it seems that GO-3 was the most suitable support and the successful immobilization of amidase on GO-3 was confirmed by TEM, elemental mapping and FT-IR spectroscopy. TEM image revealed that amidase was deposited on the surface of GO and the average size of amidase is about 5 nm (Figure 3a and Figure S10), which was consistent with its theoretical size (Figure 1a). Elemental mapping showed that C and O were homogeneously dispersed on GO surface (Figure 2b), in which the typical protein element N could also be observed. An evident band at 1545 cm^−1^ has emerged in the FT-IR spectrums of amidase@GO-3 (Figure 3c), which corresponds to N–H bending vibrations of amide II band in the infrared spectra of protein [24]. The characteristic bands of amidase at about 1650 cm^−1^ and 1545 cm^−1^ could also be observed in the FT-IR spectra of amidase immobilized on GO, GO-6 and GO-12 (Figure S11). Moreover, the existence of amidase was also confirmed by XRD and UV-vis spectrums of amidase@GO-3 biocomposite (Figures S12 and S13).

A medium modulation mechanism has been proposed to explain for the efficient immobilization of amidase on GO-3. Generally, interactions involved in enzyme immobilization on GO are electrostatic interaction and hydrophilic interaction (hydrogen bonding) due to the intrinsic property of GO [19]. The amidase used here is hydrophilic and negatively charged, as revealed by the simulation results from the Swiss Model and Discover Studio (left in Figure 3d). As GO is negatively charged in water (pH = 7.0), due to the carboxyl groups (right in Figure 3d), hydrophilic interaction turns the driving force between amidase and GO. Through surface modulation, oxygen-containing groups on GO gradually decreased and hydrophobic interaction gradually becomes the driving force. Although a higher activity could be obtained (Figure 2c) with the less negative effects of oxygen-containing groups, too-strong hydrophobic interaction will lead to a decreased enzyme activity due to the structural conformation changes, as discussed before. Thus, support GO-3, with a medium hydrophobic interaction, is the most suitable support for amidase immobilization, exhibiting the highest enzyme activity and relatively high loading capacity.

**Figure 4:**
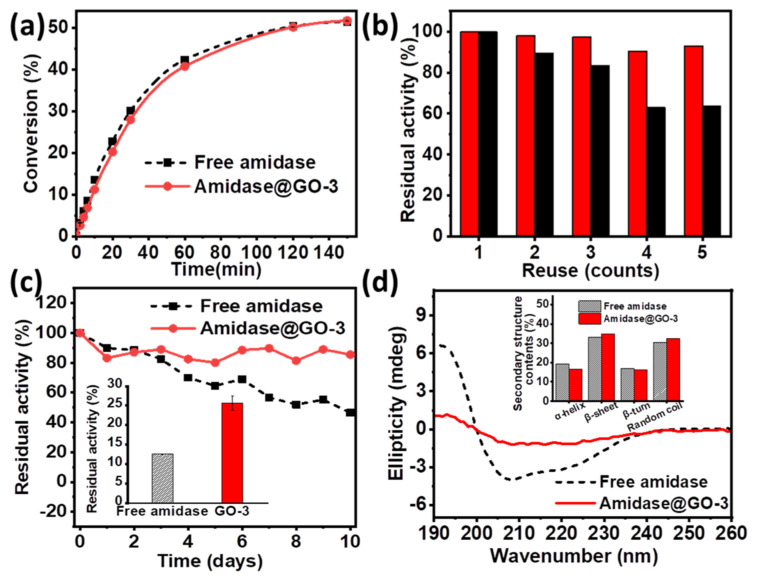
(**a**) Conversion plots of the production of L-4-fluorophenylglycine catalysed by free amidase and amidase@GO-3; (**b**) reuse of immobilized amidase on GO (black column) and GO-3 (red column); (**c**) Storage stability and high temperature tolerance (inset) of free and amidase@GO-3; (**d**) CD spectra and secondary structure contents (inset) of free amidase and amidase@GO-3.

Having demonstrated that amidase was efficiently immobilized on the surface of GO-3, biocatalytic performance of amidase@GO-3 was then investigated for biosynthesis of L-4-fluorophenylglycine. It is interesting to note that the enzymatic performance of amidase@GO-3 was similar to that of free amidase (as shown in Figure 4a), which can provide L-4-fluorophenylglycine yields of 50% within 150 min. The immobilized amidase on GO-3 maintained its 93.0% activity after five times of usage (Figure 4b), while amidase@GO only maintained 63% activity under the same condition, which can be attributed to the increased hydrophobic interaction force via surface modulation, as discussed above. Moreover, amidase@GO-3 exhibited excellent storage stability at 4 °C, which maintained its 85.5% activity after 10 days of storage, much better than that of free amidase (46.5% activity retained). Amidase@GO-3 also showed better thermal stability at 60 °C than that of free amidase (inset of Figure 4c), indicating that structural rigidity of amidase increased after immobilization on GO-3.

To further explore the reaction kinetics, apparent kinetic parameters (Michaelis–Menten) of free and immobilized amidase were investigated and compared (Table 1). The value of *K*_m_ increased and the catalytic efficiency (*k*_cat_/*K*_m_) decreased after immobilization, which might be attributed to the external mass transfer limitation by the support. Figure 4d and Figure S14 shows circular dichroism (CD) spectra of free and amidase@GO-3, suggesting the secondary structure of the amidase on GO-3 is partially lost. Secondary structure contents of β-sheet slightly increase (inset of Figure 4d), which might be explained for the increased storage and thermal stability of amidase@GO-3. These attractive properties show that the support GO-3 is an ideal candidate for amidase immobilization for the production of L-4-fluorophenylglycine.

## 4. Conclusions

In summary, it has been demonstrated that graphene oxide can serve as an efficient support for amidase immobilization, which shows the highest loading capacity (~3000 mg/g) to date. Through the modulation of GO surface via reducing the contents of oxygen-containing functional groups, an activity recovery as high as 85.3% could be achieved from amidase@GO-3. Moreover, surface-modulated GOs (including GO-3, GO-6 and GO-12) can efficiently uptake amidase under a wide range of pH values, and the maximum loading can reach ~3500 mg/g on GO-12, which can be attributed to the increased hydrophobic interaction. The resultant amidase@GO-3 biocomposites can be used as efficient biocatalysts for the synthesis of L-4-fluorophenylglycine (an intermediate of aprepitant), exhibiting good reusability and enhanced storage and thermal stability. Although the physical adsorbed amidase may fall from the material surface during operation, the ease of preparation and high amidase loading feature presented here makes the amidase@GO-3 biocomposites promising for the enzymatic biocatalysis to produce other chemicals (e.g., 6-aminopenicillanic acid) in the practical applications.

## Figures and Tables

**Table 1:** Kinetic parameters of free amidase and amidase@GO-3.

Michaelis–Menten	*k*_cat_ (s^−1^)	*K*_M_ (mM)	*V*_max_ (μmol min^−1^ mg^−1^)	*k*_cat_/*K*_M_ (10^4^ s^−1^ M^−1^)
Free amidase	4191.1	114.51	295.75	3.66
Amidase@GO-3	3322.5	127.79	234.90	2.60

## Data Availability

The data presented in this study are available on request from the corresponding author.

## Supplementary Materials

The following are available online at https://www.mdpi.com/article/10.3390/biom11101399/s1, Figure S1: SDS−PAGE of semi-purified and final purified amidase (the original western blot gel); Figure S2: XRD spectrums of the GO and graphite; Figure S3: SEM image of the GO; Figure S4: Production of L-4-fluorophenylglycine by a chemoenzymatic route using amidase (the structure in dotted box in aprepitant, the red part is the analogue of L-4-fluorophenylglycine, an intermediate); Figure S5: Conversion plots of the production of L-4-fluorophenylglycine catalysed by free amidase and amidase@GO; Figure S6: XRD spectrums of the GO, GO-3, GO-6 and GO-12; Figure S7: Conversion plots of the production of L-4-fluorophenylglycine catalysed by free amidase, amidase@GO, amidase@GO-3, amidase@GO-6 and amidase@GO-12; Figure S8: Zeta potential of amidase under different pH and the pI of amidase measured is ~4.3; Figure S9: The comparison of maximum loading capacity of amidase on GO, GO-3, GO-6 and GO-12; Figure S10: TEM image of amidase@GO-3; Figure S11: FT-IR spectrums of free amidase and amidase on different GOs (amidase@GO-3, amidase@GO, amidase@GO-6 and aidase@GO-12); Figure S12: XRD spectrums of the GO-3 and amidase@ GO-3; Figure S13: UV-Vis spectrums of the amidase, GO-3 and amidase@GO-3; Figure S14: CD spectra of free amidase, amidase@GO, amidase@GO-3, amidase@GO-6 and amidase@GO-12; Table S1: Comparison of the loading capacity of various materials reported recently; Table S2: Comparison of carbon and oxygen contents from energy dispersive spectrometer (EDS) analysis; Table S3: Influence of the enzyme loading amount on the catalytic efficiency of the immobilized amidase.
