# Supplementary material for: Surface Modulation of Graphene Oxide for Amidase Immobilization with High Loadings for Efficient Biocatalysis
Source: Biomolecules. 2021 Sep 23;11(10):1399. doi: 10.3390/biom11101399 (PMC8533581; doi:10.3390/biom11101399)
Supplement: Supplementary file 1 [file biomolecules-11-01399-s001.zip › biomolecules-1370592-supplementary.pdf]

Supplementary Material

Surface Modulation of Graphene Oxide for Amidase Immobilization with High Loadings for Efficient Biocatalysis

Kongliang Xu,[ab] Bin Wang,[ab] Chenlu Si,[ab] Chaoping Lin,[ab] Renchao Zheng*[ab] and Yuguo Zheng[ab]
[a]Key Laboratory of Bioorganic Synthesis of Zhejiang Province, College of Biotechnology and Bioengineering, Zhejiang University of Technology, Hangzhou 310014, People's Republic of China, E-mail: zhengrc@zjut.edu.cn
[b]Engineering Research Center of Bioconversion and Biopurification of Ministry of Education, Zhejiang University of Technology, Hangzhou 310014, People's Republic of China

1. Experimental Section

1.1 Amidase Expression and Purification

Recombinant amidase was expressed in *Bacillus subtilis* WB800, in which the synthesized gene (from *Bacillus megaterium* CA4098) were cloned into plasmid pPZW103 for the sequent transformation. Nucleotide sequences of amidase genes from *Bacillus megaterium* CA4098 (AF161313.1) were synthesized using the polymerase chain reaction assembly method. The synthesized genes were cloned into pGEM-T and inserted into expression vector pPZW103 between SmaI and BamHI restriction endonuclease sites. Ligated plasmid pPZW103 was transformed into B. subtilis WB800. For the selection of B. subtilis WB800 transformants, 50 μg/mL kanamycin was added to the LB medium. DNA manipulation, plasmid isolation, and agarose gel electrophoresis were performed according to the standard protocols.

B. subtilis WB800/pPZW103-PGA was cultivated on LB agar medium containing 50 μg/mL kanamycin at 37 °C for 20 h. The colony was picked and inoculated in 60 mL of LB medium containing 50 μg/mL kanamycin in a 500 mL flask. After incubation at 37 °C and 150 rpm for 12 h, 2% (v/v) of the seed cultures was inoculated with 3 L of fermentation medium [10.0 g/L soluble starch, 12.0 g/L peptone, 3.0 g/L yeast extract, and 10.0 g/L sodium chloride (pH 7.5)] in a 5 L fermentor. Fermentation was conducted at 37 °C, 0.5 vvm, and 150 rpm. After being cultivated for 24 h, the supernatant was harvested by centrifugation (12000g and 4 °C) for 10 min.

Ammonium sulfate was slowly added to the supernatant to 80% saturation while the mixture was being stirred at 0 °C. The precipitate was harvested and dissolved in sodium phosphate buffer (0.05 M, pH 7.8). The solution was dialyzed overnight with the same buffer. The dialyzed solution was loaded on an anion ionexchange column (DEAE-IEX, 5 mL, Bio-Rad Laboratories) using a mobile phase (0.05 M, pH 7.8 phosphate buffer) at a flow rate of 1.0 mL/min. The anion ion-exchange column was eluted with a linear gradient of 0 to 1.0 M sodium chloride buffer. Active PGA fractions were supplemented with ammonium sulfate to a final concentration of 2.0 M by being stirred. The resulting solution was loaded on a hydrophobic interaction chromatography column (Butyl HIC, 10 mL, Bio-Rad Laboratories), which was equilibrated with 20 mM sodium phosphate buffer [2.0 M ammonium sulfate in the buffer (pH 7.2)]. The PGA was then eluted by a linear gradient from 2.0 to 0.6 M ammonium sulfate in 20 mM sodium phosphate buffer (pH 7.2) at a flow rate of 1.0 mL/min. The active PGA fractions were collected and dialyzed. The protein purity was determined by SDS−PAGE. The protein concentration was measured by the Bradford method using the protein assay kit (Bio-Rad Laboratories) with bovine serum albumin as a standard.

1.2 Preparation of GO

GO was prepared using natural graphite flakes by a modified Hummers method. In a typical procedure, 0.75 g of $NaNO_3$ was added to 34 mL of H_2SO_4. Subsequently, 1 g of graphite flake was added to the solution at 0 °C and then the mixture was oxidized by 5 g of $KMnO_4$ with vigorous mixing. After increasing the temperature to 35 °C, the solution was stirred more for 2 h. The mixture was diluted by 50mL of distilled water at 0 °C, and mixed for 2 h. Then, 4 mL of H_2O_2 was slowly added for oxidization and stirred vigorously until gas was not generated. The mixture was then centrifugated and washed with 1:10 aq. HCl (500 mL) and DI water (500 mL). To completely wash the graphite oxide flakes, the resultant graphite oxide was dissolved in water again and centrifugated several times to reach the pH 7. The graphite flakes were sonicated with 70 W power in water for 5 h, and finally centrifuged at 4000 rpm to collect a GO solution from a supernatant.

1.3 Surface Modulation of GO

The contents of oxygen-containing groups on the GO surface was controlled by L-ascorbic acid. The degree of reduction was controlled by the reaction time. Briefly, the reduction reaction was performed by adding l-AA (200 mg) to an aqueous dispersion of the GO (2 mg mL^{-1}, 50 mL). After the reduction reaction was complete, the solid was isolated by filtration and washed with distilled water until the pH of the solution was 7.0.

1.4 Immobilization of Amidase on GO

Enzyme immobilization was carried out by adding the desired amount of GO to a 0.1 M phosphate buffer (pH=7.0) of amidase at 4 °C, affording the bio-composite denoted as amidase@GO. The mixture was incubated for 1 hour with shaking and then centrifuged. The supernatant was used to determine the enzyme loading. The immobilized enzymes were washed three times with the same buffer to remove physical adsorbed enzymes. The loading capacity of GO for amidase was obtained via a bicinchoninic acid (BCA) assay using UV−vis spectroscopy to detect the concentration of amidase in the supernatant solutions before and after immobilization

1.5 Material characterization/Instruments

Scanning electron microscopy (SEM) images were acquired on JSM-7610F scanning electron microscopy (SEM) (Hitachi, Japan) and Gemini SEM 300 (Zeiss, German). Transmission electron microscopy (TEM) images were taken on a JEM-1010 transmission electron microscope (Hitachi, Japan). Powder X-ray diffraction (XRD) patterns were collected using an X'TRA deffractometer (ARL, Switzerland). Samples were prepared by dispersing 30 mg of sample on a flat, glass plate PXRD holder with a diameter of 2.5 cm. A stretched piece of plastic film was then used to spread, flatten and hold the sample in position for measurement. Fourier-transform infrared spectroscopy (FT-IR) characterization was obtained on a Thermo Nicolet 380 spectrometer using KBr pellets (Wisconsin, USA) and FTIR spectra were collected from 500 cm^{-1} to 4000 cm^{-1}.

2 Figure S1-S14

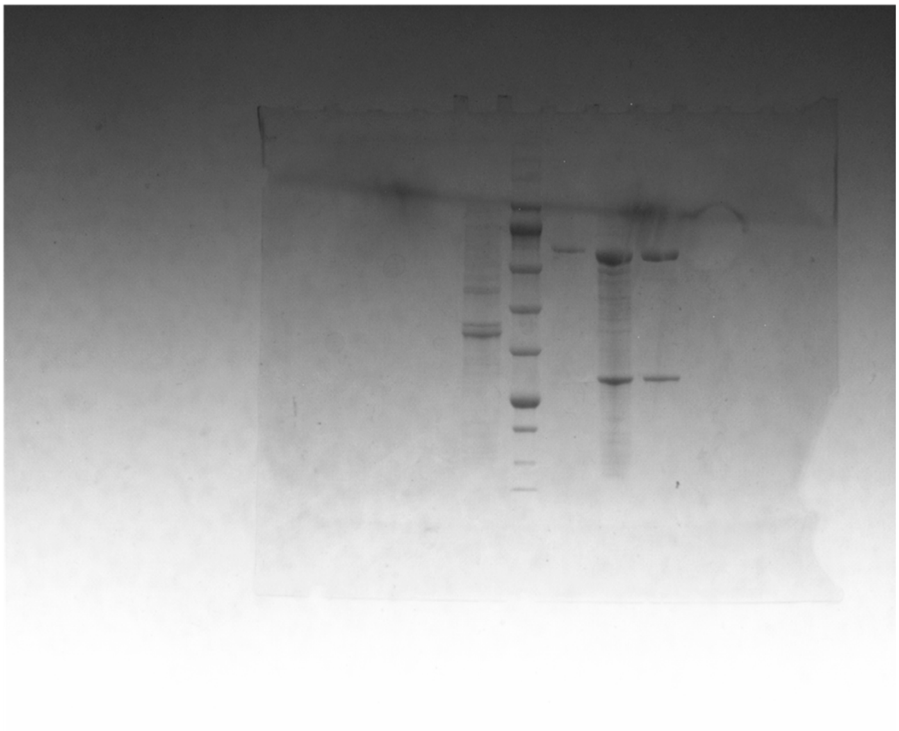

Figure S1. SDS−PAGE of semi-purified and final purified amidase (the original western blot gel)

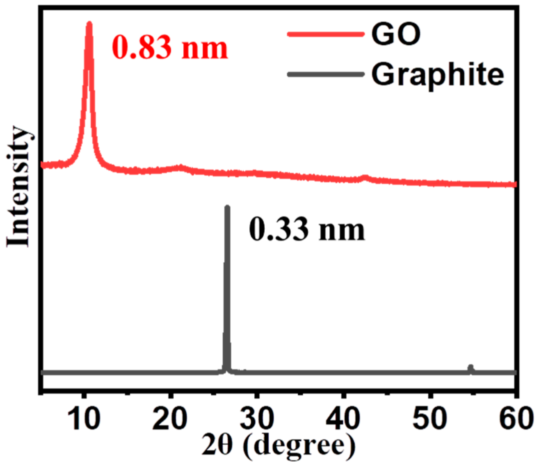

Figure S2. XRD spectrums of the GO and graphite.

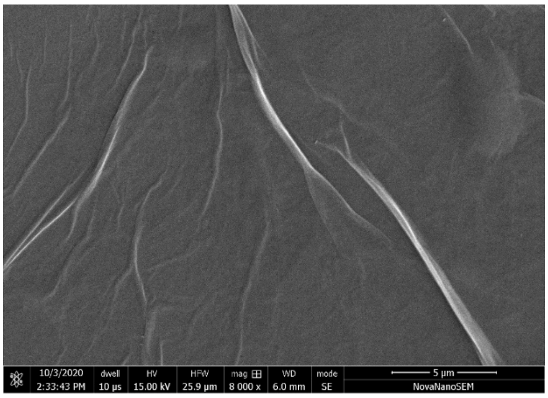

Figure S3. SEM image of the GO

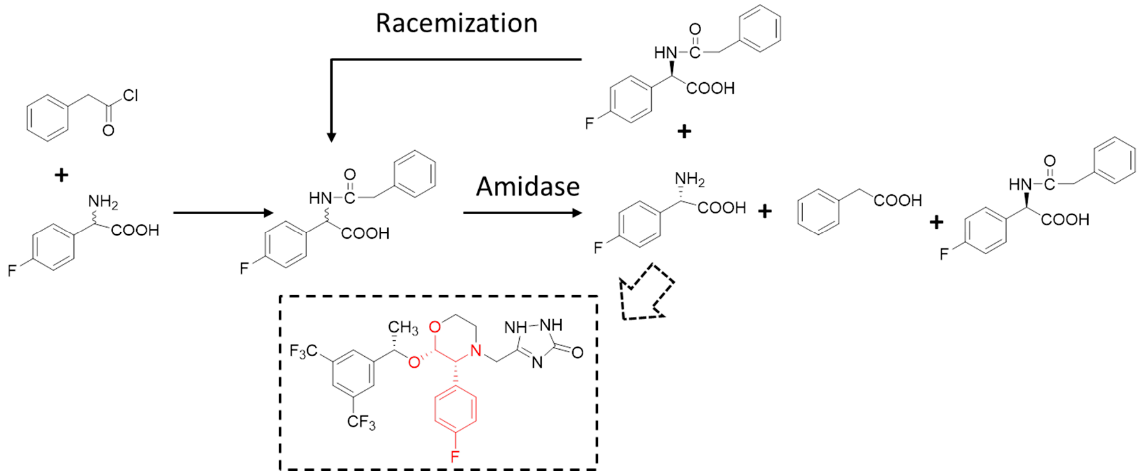

Figure S4. Production of L-4-fluorophenylglycine by a chemoenzymatic route using amidase (the structure in dotted box in aprepitant, the red part is the analogue of L-4-fluorophenylglycine, an intermediate)

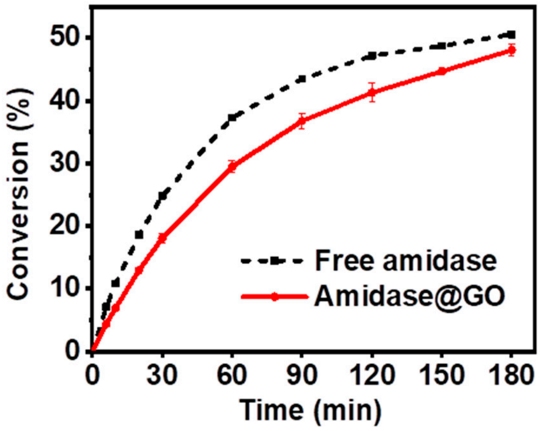

Figure S5. Conversion plots of the production of L-4-fluorophenylglycine catalysed by free amidase and amidase@GO.

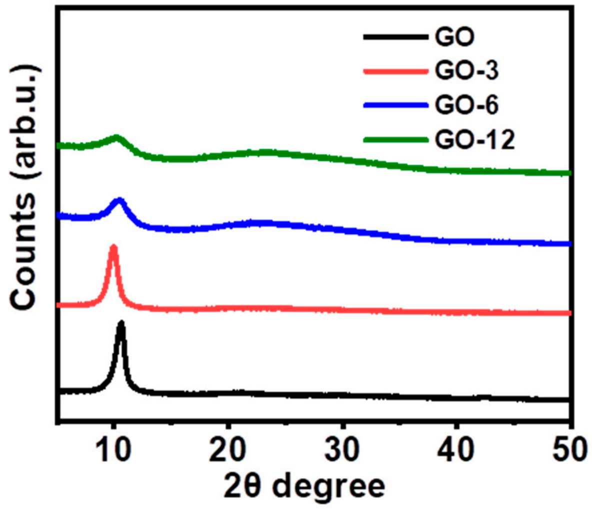

Figure S6. XRD spectrums of the GO, GO-3, GO-6 and GO-12.

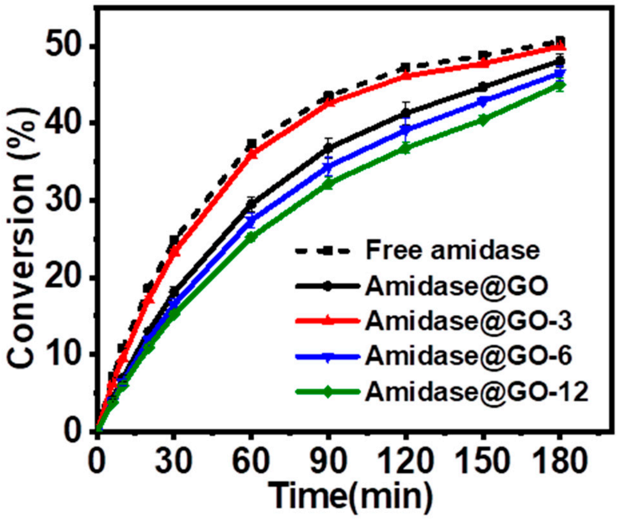

Figure S7. Conversion plots of the production of L-4-fluorophenylglycine catalysed by free amidase, amidase@GO, amidase@GO-3, amidase@GO-6 and amidase@GO-12.

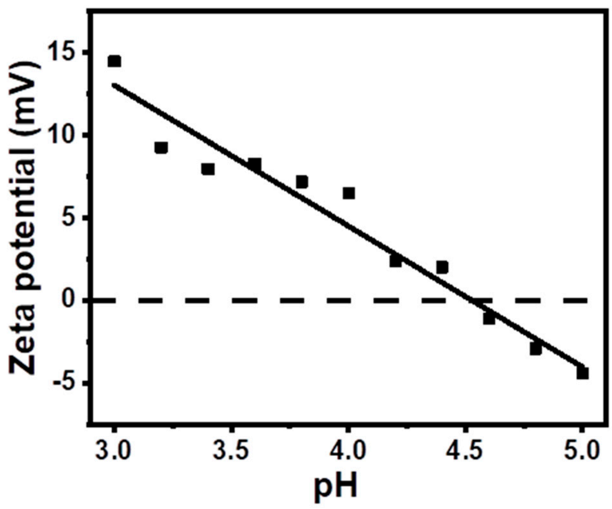

Figure S8. Zeta potential of amidase under different pH and the pI of amidase measured is ~4.3.

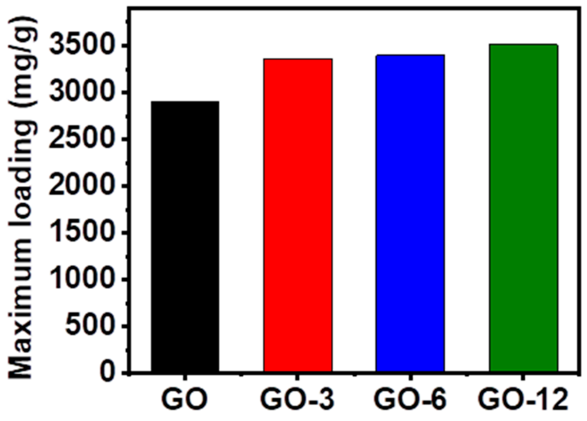

Figure S9. The comparison of maximum loading capacity of amidase on GO, GO-3, GO-6 and GO-12.

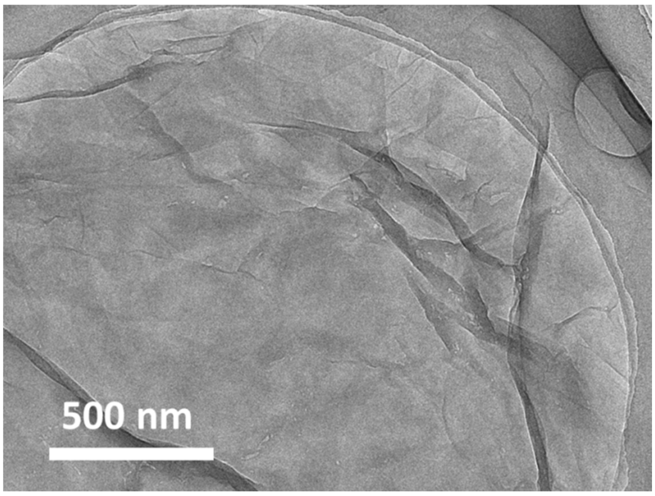

Figure S10. TEM image of amidase@GO-3

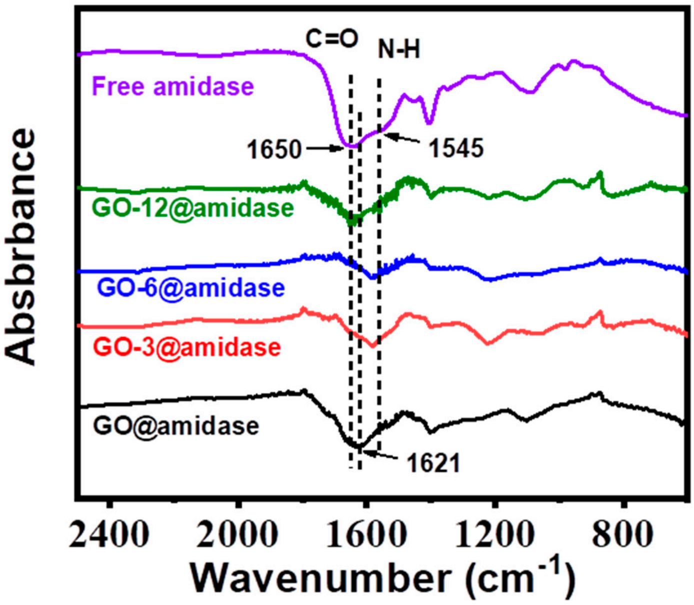

Figure S11. FT-IR spectrums of free amidase and amidase on different GOs (amidase@GO-3, amidase@GO, amidase@GO-6 and aidase@GO-12)

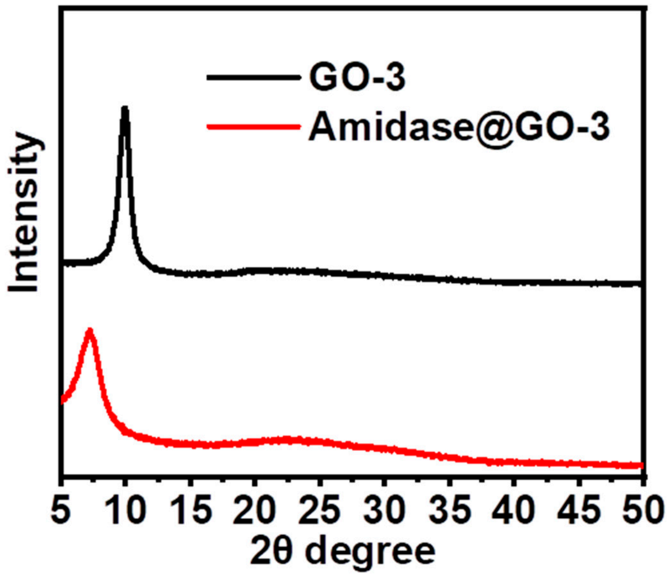

Figure S12. XRD spectrums of the GO-3 and amidase@ GO-3.

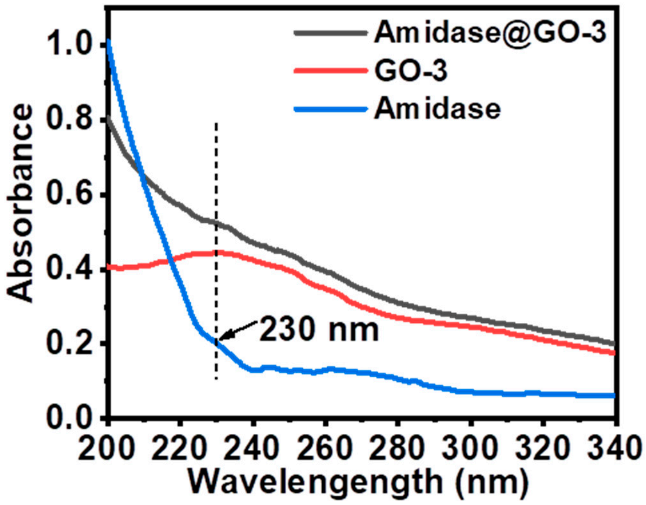

Figure S13. UV-Vis spectrums of the amidase, GO-3 and amidase@GO-3.

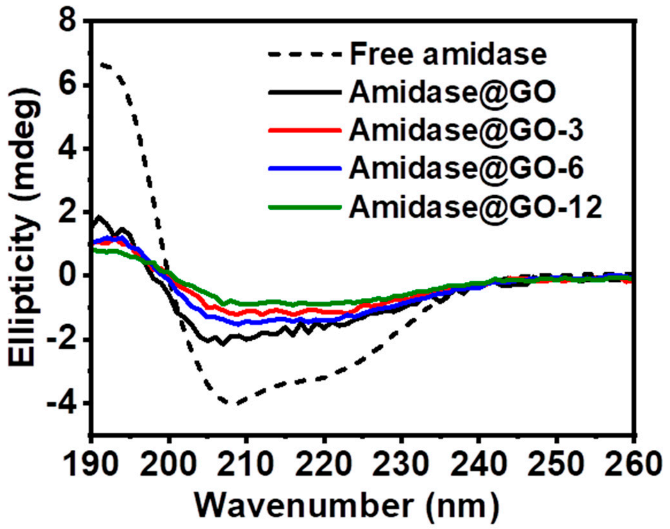

Figure S14. CD spectra of free amidase, amidase@GO, amidase@GO-3, amidase@GO-6 and amidase@GO-12.

3 Table S1-S3

Table S1 Comparison of the loading capacity of various materials reported recently.

Materials	Enzyme	Maximum Loading	Products	Ref.
Graphene Oxide	Amidase	2902 mg/g	L-4-fluorophenylglycine	This work
Macro-mesoporous silica spheres	Penicillin G Amidase	895 mg/g	6-aminopenicillanic acid	1
Mesocellular siliceous foams	Penicillin G Amidase	555.0 mg/g	6-aminopenicillanic acid	2
Copolymer	Penicillin G Amidase	201 mg/g	6-aminopenicillanic acid	3
Fe_3O_4 microparticles	Penicillin G Amidase	374 mg/g	6-aminopenicillanic acid	4
Silica nanoflower@metal-organic framework	Penicillin G Amidase	382.3 mg/g	6-aminopenicillanic acid	5
UiO-66-NH_2	Amidase	211.2 mg/g	L-4-fluorophenylglycine	6
Magnetic nanocrystalline cellulose	Penicillin Amidase	172.3 mg/g	7-amino-3-deacetoxyceph alosporanic acid	7
Epoxy-activated Sepabeads	Cephalosporin C Amidase	200-300 mg/g	7-aminocephalosporanic acid	8

Table S2 Comparison of carbon and oxygen contents from energy dispersive spectrometer (EDS) analysis.

	C(%)	O(%)
GO	54.41	45.59
GO-3	54.59	44.43
GO-6	59.04	40.9
GO-12	59.59	40.68

Table S3 Influence of the enzyme loading amount on the catalytic efficiency of the immobilized amidase.

Column	Enzyme loading amount	Apparent activity (U)	Specific activity (U/mg)
amidase@GO-3	2000 mg/g	367.16	183.58
amidase@GO-3	900 mg/g	172.81	192.02
amidase@GO-3	170 mg/g	39.93	234.90

4 References

1. J. Q. Zhao, Y. J. Wang, G. S. Luo and S. L. Zhu, *Bioresour. Technol.*, 2011, **102**, 529-535.
2. H. Shi, Y. Wang and G. Luo, *Ind. Eng. Chem. Res.*, 2014, **53**, 1947-1953.
3. K. Li, G. Shan, X. Ma, X. Zhang, Z. Chen, Z. Tang and Z. Liu, *Colloids Surf. B*, 2019, **179**, 153-160.
4. X. Li, L. Tian, Z. Ali, W. Wang and Q. Zhang, *J. Mater. Sci.*, 2018, **53**, 937-947.
5. Y. J. Du, J. Gao, H. J. Liu, L. Y. Zhou, L. Ma, Y. He, Z. H. Huang and Y. J. Jiang, *Nano Res.*, 2018, **11**, 4380-4389.
6. C. P. Lin, K. L. Xu, R. C. Zheng and Y. G. Zheng, *Chem. Commun.*, 2019, **55**, 5697-5700.
7. Z. X. Huang, S. L. Cao, P. Xu, H. Wu, M. H. Zong and W. Y. Lou, *Chem. Eng. J.*, 2018, **346**, 361-368.
8. C. Boniello, T. Mayr, I. Klimant, B. Koenig, W. Riethorst and B. Nidetzky, *Biotechnology and Bioengineering*, 2010, **106**, 528-540.
